# Digital transformation of the harm reduction sector—“Here4UScotland” a case study of a virtual supervised consumption

**DOI:** 10.1177/20552076251390561

**Published:** 2026-01-27

**Authors:** Hadi Daneshvar, Graeme Strachan, Catriona Matheson

**Affiliations:** 1 School of Health and Social Care, 3121 Edinburgh Napier University - Sighthill Campus, UK; 2 School of Law, 7315 The University of Sheffield, UK; 3 Centre for Healthcare and Community Research, 7622 University of Stirling, Scotland, UK

**Keywords:** Digital transformation, digital health, drug-related death, harm reduction, remote supervised consumption

## Abstract

**Objective:**

This study explores the potential of digital technologies in reducing drug-related deaths through virtual supervised drug consumption. It assesses barriers, enablers, and strategies for adopting a remote supervision service app, Here4UScotland, fostering user engagement and ownership.

**Methods:**

A mixed-methods evaluation was undertaken, using semi-structured interviews, focus groups, and quantitative data. Interviews and focus groups were undertaken with 26 participants. The Technology, People, Organizations, and Macro-environmental framework guided data collection and analysis, while the Transformative Technology Integration in Health conceptual model enabled analysis of the relationship between digital technology and service delivery. Qualitative data were thematically analyzed.

**Results:**

The app was piloted in Aberdeen (Scotland) from January to December 2023. Twenty-five users received smartphones and logged 74 calls. Qualitative findings identified user concerns about privacy versus the need for real-time support, challenges in integrating new features, and the impact of police involvement on trust in digital services. The app's functionality and user engagement highlighted the need for ongoing support and improved system integration. Interviews highlighted the importance of relationships, training, and strategic outreach in successfully delivering digital harm reduction services. Technological features, such as location tracking, offer real-time support but raise privacy concerns.

**Conclusion:**

Organizational and macroeconomic factors, including marketing, outreach, and law enforcement involvement, may impact service effectiveness and should be considered in future app implementations. Despite challenges, digital tools have enhanced accessibility and support in overdose prevention. Future research should explore cultural differences in digital adoption and improve communication strategies to maximize user engagement.

## Introduction

Recent data underscores the significant impact of drug use on mortality rates. A 2024 report from the World Health Organization indicates that psychoactive drug use is responsible for ∼600,000 deaths annually.^1^ In 2023, the United States recorded 105,007 drug overdose deaths (ODs), resulting in an age-adjusted rate of 31.3 deaths per 100,000 individuals.^2^ Canada reported 7328 ODs in 2022.^3^ Within the European Union, over 6000 drug-related deaths (DRDs) were reported in 2021.^1^ In Scotland, 1172 DRDs were registered in 2023, marking a 12% increase from the previous year.^4^ This figure underscores Scotland's position as having the highest OD rate in Europe, with 27.7 deaths per 100,000 people, nearly three times higher than the next highest country, Ireland.

People who use drugs, especially those at risk of OD, often use alone, increasing the risk of fatal OD.^5–7^ Opioid overdoses can be reversed with the timely administration of naloxone, an effective opioid antagonist.^8^ Laypersons can safely administer it as a nasal spray, making it accessible for public use. Distributing naloxone is a key strategy in combating the opioid epidemic internationally,^9^ with Scotland investing significantly in increasing access for people who use drugs and their personal support networks.^10^

The global healthcare landscape has been significantly transformed by digital technologies, facilitating the development and implementation of numerous health interventions. Digital technology encompasses electronic and computational tools, systems, or platforms that facilitate information exchange, communication, or service provision through digital or virtual means.^11^ Specifically, within the context of harm reduction explored here, digital technology refers explicitly to the smartphone application. The World Health Organization's Global Strategy on Digital Health 2020–2025 emphasizes the potential of digital health solutions to enhance health outcomes and service delivery worldwide.^12^ Innovations such as telemedicine, wearable health monitors, and artificial intelligence (AI)-driven diagnostics are revolutionizing patient care, improving access, and increasing efficiency.^13^

Recent scholarship underscores both the promise and pitfalls of digital platforms in supporting overdose prevention. Reveal that social media-based drug networks often rely on established peer ties, which can reduce overdose risks by ensuring safer, more reliable supply chains. Gomes and Sultan^14^ caution that algorithmic content moderation on platforms like Instagram and TikTok may unintentionally remove vital harm-reduction messages—such as naloxone guidance or overdose warning alerts—thereby limiting their preventive potential. Additionally, Volpe et al.^15^ illustrate how digital drug-alert systems struggle under “prohibition-world” regulations, hampering real-time overdose alert deployment. Together, these findings highlight the need for digital overdose-prevention tools—like virtual supervised consumption apps—to be thoughtfully designed to leverage peer networks while navigating moderation constraints and regulatory environments. While digital solutions have been widely adopted across various healthcare sectors, their integration into substance use treatment and harm reduction has been comparatively slower. Nonetheless, emerging evidence indicates that digital technologies can play a significant role in reducing overdose fatalities and enhancing care for individuals who use drugs. For instance, the Digital Lifelines Scotland (DLS) program has demonstrated that providing digital devices and internet access to people who use drugs can improve connectivity, access to services, and overall well-being. Importantly, DLS also highlighted how anonymity, person-centered outreach, and digital inclusion can foster trust and engagement among vulnerable users, thereby contributing to harm reduction efforts.^16^ Similarly, a qualitative study highlighted that digital inclusion initiatives can enhance support within harm reduction services, though challenges such as technical knowledge gaps and organizational constraints need to be addressed for successful implementation.^17^ Furthermore, research has shown that online resources, including forums and therapeutic platforms, are valuable tools in substance misuse recovery, offering accessible support and information to individuals at various stages of their recovery journey.^18^

Digital harm reduction refers to the adaptation of traditional harm-reduction strategies—providing resources, education, and support to people who use drugs—into digital formats such as mobile apps, online communities, and virtual spotting services. This approach leverages anonymity, real-time connection, and low-threshold access to deliver tools for overdose monitoring, safer-use guidance, peer support, and emergency interventions, particularly for opioid and stimulant users. A recent scoping review of “electronic harm reduction” interventions (including apps, hotlines, and virtual spotting services) emphasizes their emerging potential to prevent opioid-related ODs, though evidence is still developing.^19^

Drug consumption rooms are supervised facilities that reduce ODs and other harms. More than 100 operate internationally, and the first legal site in the UK, The Thistle, opened in Glasgow in 2025.^20–22^ In addition to physical drug consumption rooms, virtual supervised consumption initiatives are emerging as innovative approaches to harm reduction. International evidence highlights the success of virtual methods during COVID-19. A study by Perri et al.^23^ trialed an informal spotting method where users called friends while using drugs, which provided remote supervision and support. This approach reduced risks of harm, criminalization, and stigma, and offered overdose prevention for non-injecting users. For example, the US national “Never Use Alone”^24^ program, Massachusetts' SafeSpot^25^ program, and Scotland's “We Are With You” helpline all offered phone-based supervision, prioritizing privacy and safety.^26^

Daneshvar et al.^27^ compared responder apps for overdose prevention that require active user check-ins during drug use. These apps monitor users and contact supporters or emergency centers if needed. Two functions of interactive response apps are, firstly, the user activates the app before drug use; if they do not respond to an alert after a set period, the app connects to a supporter or emergency center (e.g. Lifeguard, OB Buster, and UnitiPhily). Secondly, the app provides a platform for users to communicate and live talk with a trained supporter until they are safe, enabling a rescue plan if needed or connecting to emergency services (e.g. Brave). Internationally, the Brave app, based in Canada,^28^ provides remote supervision and anonymous peer support for people, functioning as a 24/7 remote supervised consumption space with access to emergency response. This app was terminated in January 2025. Following learning from the Daneshvar et al.^27^ and Oteo et al.^28^ study, Brave was selected for a 12-month pilot in Aberdeen City after evaluating all available apps based on implementation strategy, cost-effectiveness, and organizational readiness. This collaborative project involved the University of Stirling (lead and evaluator), Alcohol and Drugs Action (ADA—a local harm reduction service—provided and trained supporters from their staffs and promoting the app to its service users), Brave Technology Co-op, Vancouver, Canada (app developer and technical support), and the Digital Health and Care Innovation Centre (DHI-Scotland—provided regulatory support). Funding was provided by the DLS program, supported by the Scottish Government. Aberdeen City was selected because it suffered a similar increase in ODs as observed across the rest of Scotland (National Records of Scotland, 2021), and the structure of the city and its facilities were accessible (e.g. ADA). The app was given a new name, “Here4UScotland,” through a user-driven co-design process to enhance localization. The Here4UScotland app was functionally similar to the Brave app but was localized to the Scottish context, with adaptations in documentation and integration with local emergency services. It provided anonymous, peer-to-peer supervision during drug consumption. Upon activation by the caller, individuals who use drugs were connected via voice to a trained “Supporter,” with whom they agreed upon a personalized rescue plan and remained in communication throughout consumption. If the caller became unresponsive, the Supporter activated an agreed-upon emergency response utilizing the user's location. This response involved contacting a designated “Rescuer,” who was pre-identified within the rescue plan, either to administer naloxone—freely accessible in Scotland—or to engage the ambulance service. ADA, a local harm reduction service in Aberdeen, hosted the application and recruited supporters from among their staff, who then received targeted training. Callers were recruited from ADA's existing service users, with the app promoted during routine service contacts, including harm reduction drop-ins, outreach activities, and one-to-one support sessions. Recruitment was, therefore, directed at individuals already engaged with ADA services, rather than through general street-based outreach or flyer distribution.

## Objective

Digital technology can support, assist, and enhance both current and innovative new services.^11^ This article examines the potential of digital transformation and the provision of technology for people who use/d drugs (called service users hereafter) and identifies barriers and enablers in the use of digital technologies for preventing DRDs. The study objectives were to:

1. Examine the impact of virtual access to remote supervision via a smartphone app on those using drugs, and on those providing support in the Scottish context.
2. Evaluate the possibilities for evolving and adapting the app and service to further expand digital solutions to reduce DRDs.
3. Assess how digital supervision can be transformed into a model that encourages service user adoption through positive interaction and engagement, thereby fostering individual and collective ownership.

## Method

### Study design

This article draws data from a broader study that evaluated the Here4UScotland app through a mixed-methods approach that included semi-structured qualitative interviews, focus groups, and quantitative data.^29^ The quantitative data provided by Brave offered a broad view of the application's usability, while the qualitative data provided deeper insights into user experiences and underlying perceptions.

### Theoretical frameworks

**Figure 1.**
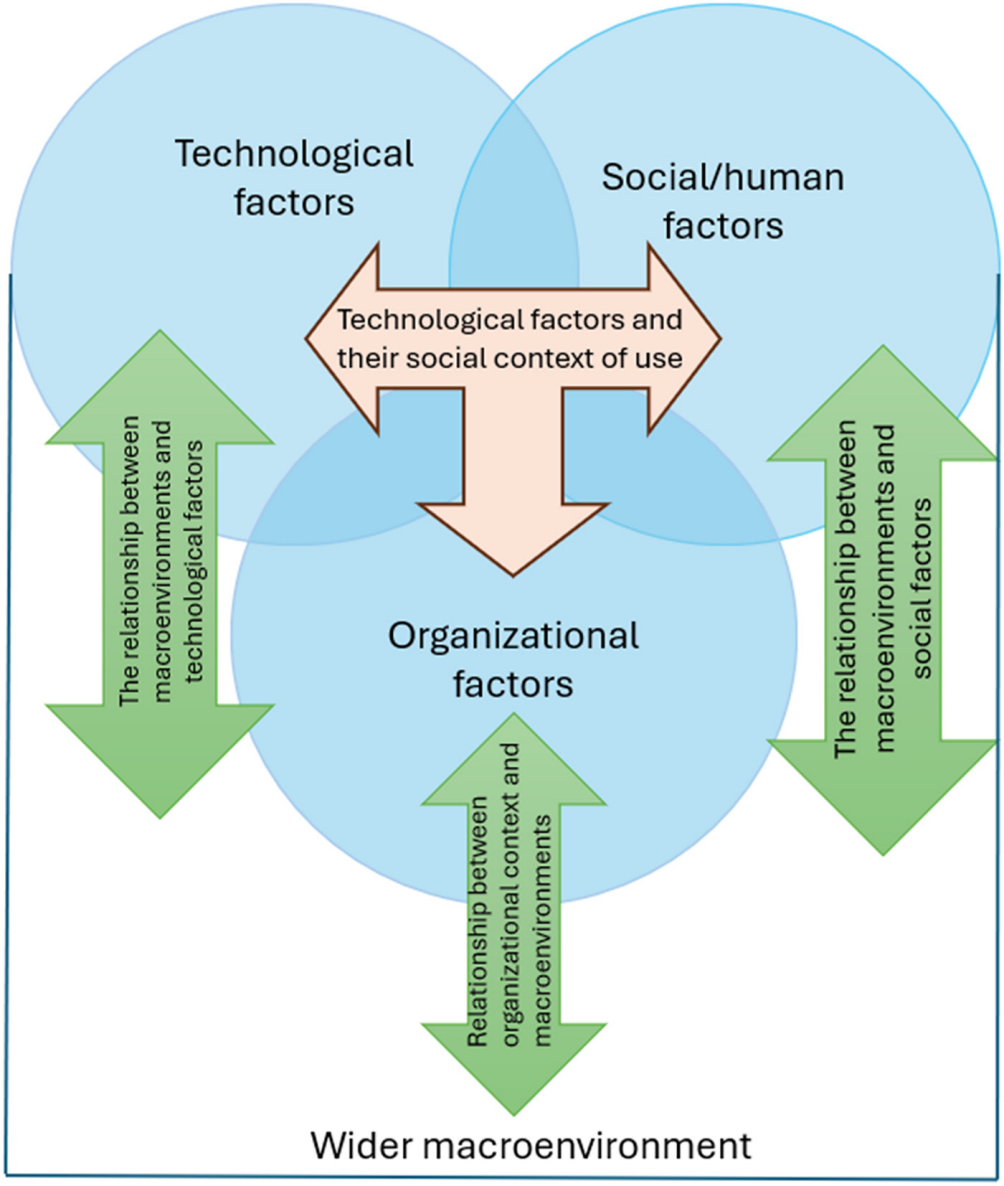
Diagram of the Technology, People, Organizations, and Macro-environmental (TPOM) evaluation framework.

The study used two frameworks. First, we have used the Technology, People, Organizations, and Macro-environmental (TPOM)^30,31^ framework to navigate the complex implementation landscape for health technologies. This framework focuses on technological, social, organizational, and macro-environmental factors,^30^ making it well-suited for assessing technology in complex healthcare and social care settings.^16,17,32^ Additionally, TPOM's formative approach supports continuous evaluation and refinement throughout the implementation stages. Figure 1^30^ illustrates the TPOM framework, which was used in data collection and data analysis as explained in the next sections.

**Figure 2.**
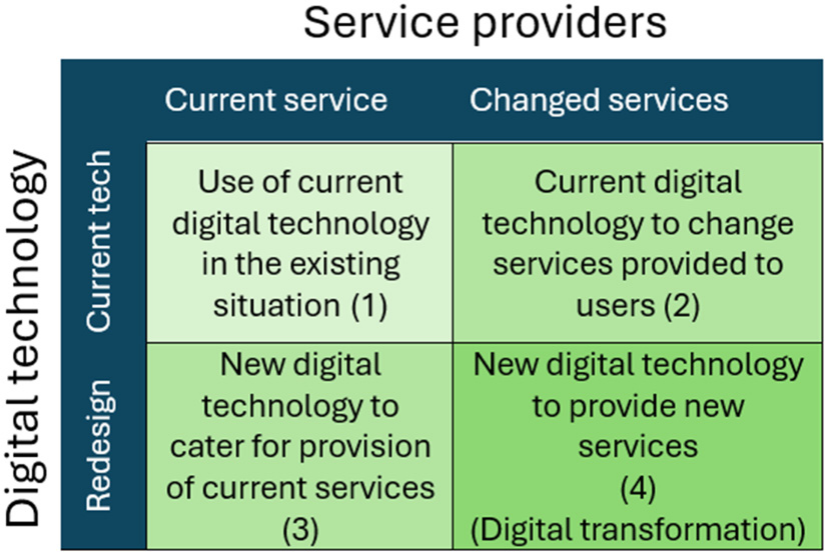
Transformative technology integration in health (TTIH) conceptual framework.

Secondly, in order to analyze and explore the relationship between digital technology and the provision of services, the Transformative Technology Integration in Health (TTIH- framework was named in this study.) conceptual framework developed by Daneshvar et al.^17^ was applied. The TTIH model has two aspects: incorporating digital tools into the delivery of current services and developing new services by adopting and utilizing established or emerging digital technologies (Figure 2). This framework provides a model for assessing digital transformation within service providers. It operates on two axes: the service itself (current vs. new/emerging) and the digital technology applied (existing vs. emerging). The core purpose is to evaluate how the integration of either new or existing digital technologies enables service providers to innovate, reshape their services, and transform their operations.

TPOM captures four interrelated domains—technological aspects (e.g. usability and interoperability), social and human factors (e.g. trust and engagement), organizational issues (e.g. training and workflows), and macro-environmental influences (e.g. policy and regulation). This structure enabled us to systematically map barriers and enablers across multiple layers of the implementation context.

However, TPOM primarily addresses the context of implementation rather than the transformative potential of technology itself. To complement this, we applied the TTIH model. TTIH differentiates between current versus emerging services and existing versus emerging technologies, thus allowing us to position Here4UScotland both as a digitalization of an existing harm-reduction service (remote supervision) and as a catalyst for new forms of support (e.g. anonymized peer-to-peer supervision and data-informed outreach).

Using both frameworks in combination was methodologically necessary. TPOM allowed us to evaluate how the technology was implemented and received, while TTIH allowed us to evaluate what kind of service transformation resulted from the implementation. Together, the frameworks provided a more comprehensive understanding of both the process and the outcomes of digital transformation in harm reduction. We refer back to this combined evaluation in the Discussion section to show how contextual and transformational perspectives jointly informed our interpretation of findings.

### Qualitative data

The qualitative components of this study were reported in line with the Consolidated Criteria for Reporting Qualitative Research (COREQ) checklist to ensure methodological rigor and transparency.^33^

#### Participants and requirements

Qualitative data were collected from three groups: service users (callers) included individuals aged ≥ 18 years old, currently using or having used illicit drugs in the past 12 months, and either using or having been offered the app while residing in Aberdeen. Service providers (supporters) were community members trained in harm reduction, aged ≥ 18, and designated to assist app users. Community stakeholders encompassed frontline staff, managers, responders, and volunteers from organizations providing harm reduction and community outreach, as well as local policymakers, Scottish Ambulance Service (SAS) harm reduction leads, and the police.

#### Data collection

The interview guide was developed by HD and CM (see Supplemental Material 1), incorporating relevant areas of the TPOM and drawing on insights from the broader Here4UScotland delivery work and a prior user need study.^29^ The topic guide for callers focused on app usability and the social/human (people) domains, examining the service's usage, any usability issues, and its effects on them. For supporters, the emphasis was on infrastructure, training, usability, support needs, and the impact of their relationships with service users. Community stakeholders focused on examining the app's impact, identifying gaps, barriers, and enablers at organizational and macro-environmental levels, including sustainability. The focus group guide developed for community stakeholders was used as an interview guide with this group.

Initially, we offered all callers the option of a focus group or an interview to maximize participation. However, due to the individual privacy concerns of callers—particularly those recruited through ADA—the planned caller focus group did not take place. Supporters were offered either a focus group or an interview. It was always planned to use a focus group for community stakeholders because they are participating at a higher/organizational level with less privacy concerns. However, the interview option was also available, and a number of stakeholders chose this option. Overall, it was a combination of maximizing participation and being efficient with time in a manner that suited different groups. Interviews and focus groups were conducted from May to November 2023. Participants were provided with an information sheet and had the opportunity to ask questions. Written or verbal informed consent was provided before each interview and focus group. Written consent was obtained when participants were able to meet in person or had access to electronic means to sign and return consent forms. Verbal consent was used in situations where participants were interviewed remotely (e.g. by telephone or online), or when logistical constraints made it impractical to obtain written documentation. In all cases, the consent process followed ethical guidelines and was approved by the institutional review board.

All caller and supporter participants were recruited by ADA^34^ staff. Interviews were arranged via email, phone, or in person. All interviews took place either in person, on MS Teams, or by phone with the researcher (GS). Callers received a £10 voucher as an honorarium. All callers were provided with a debrief sheet that outlined information about the study and sources of support.

Two separate focus groups with supporters were conducted by GS and HD on Microsoft Teams, whilst an in-person focus group with stakeholders took place in Aberdeen, led by CM, HD, and GS. GS invited the supporters, and CM recruited the stakeholders. Focus groups with supporters were recruited through ADA, and stakeholders were invited by email from CM. We stopped the data collection when saturation was reached, with no insights emerging.

The authoring team comprised members of mixed gender, all of whom are educated to the Master's or PhD level and have extensive experience working with people who use drugs and service staff. GS has relevant lived experience.

Interviews provided in-depth, personalized perspectives from individual service users and supporters in a confidential environment, ideal for exploring sensitive experiences, privacy concerns, usability issues, and subjective impacts of the app. Focus groups were particularly suited to examining shared experiences, systemic barriers, enablers, and collaborative insights from community stakeholders and supporters, where discussions were at a macro-environment and organizational level.

### Quantitative data collection

To evaluate the app's technical acceptability, quantitative data on app downloads and monthly call volumes were supplied by Brave, the app developers.

### Data analysis

Data analysis consisted of three phases: (1) descriptive quantitative analysis of uses of the app by its users; (2) evaluation of the app based on the TPOM framework; and (3) understanding the relationship between digital technology and the provision of services using the TTIH framework.

In Phase 1, quantitative data were used to generate frequency tables using Microsoft Excel. Simple descriptive statistics were used to describe the uptake and the level and nature of use.

Then, interviews and focus groups were transcribed verbatim by an external transcriber, with identifiable information removed; Scottish dialects were preserved to preserve the authenticity of the participants’ language. All data and quotes were pseudonymized, with each attributed to a participant while omitting identifiable names, locations, or other personal details. NVivo (version 12) was used for qualitative data analysis.^35^

**Figure 3.**
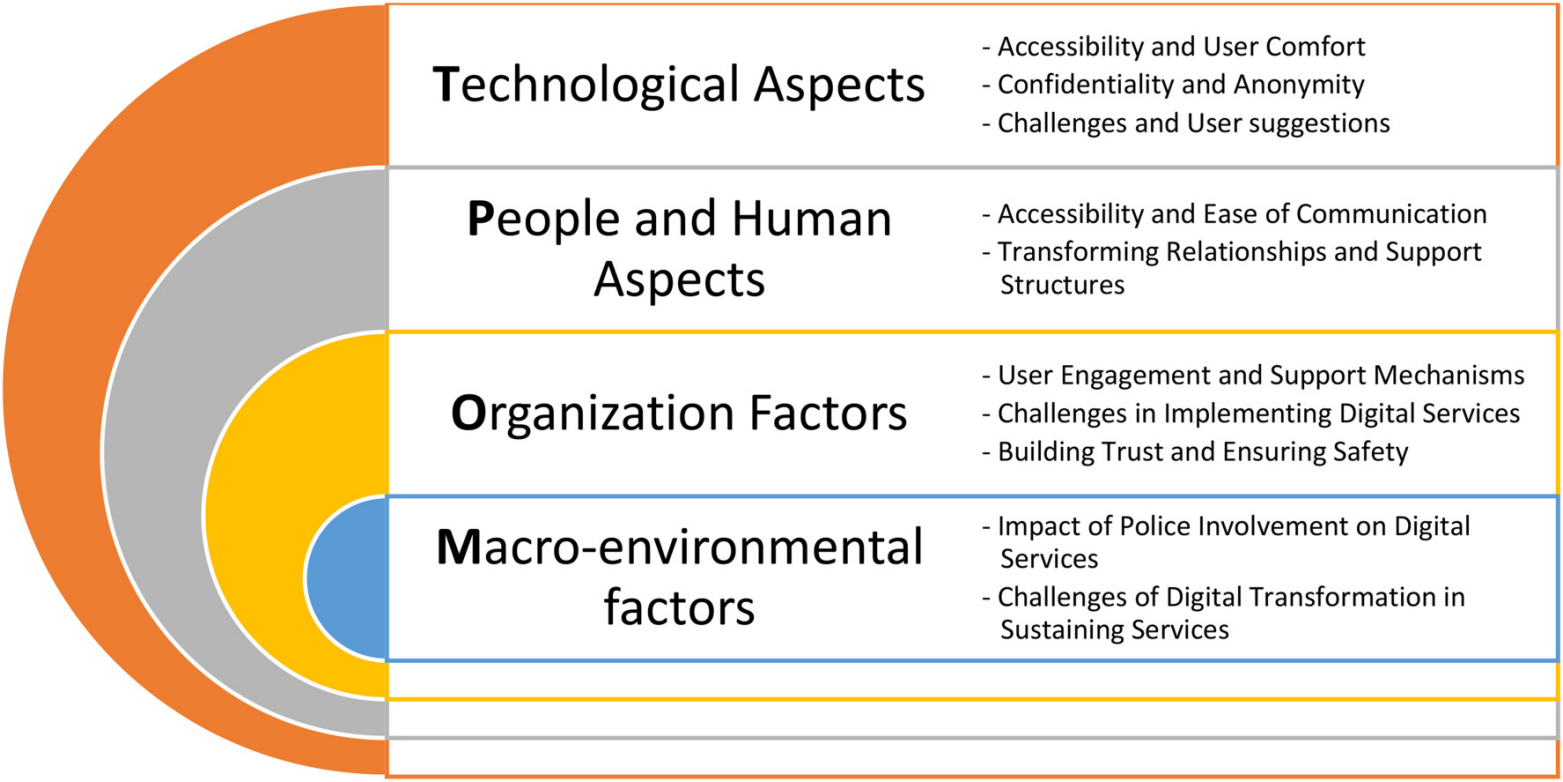
Key themes and subthemes.

In Phase 2, the TPOM was used as a conceptual framework for analysis.^30^ We applied thematic analysis^36^ to our data first to organize the findings based on the four dimensions of the TPOM framework, and then formed subthemes within each dimension. This phase involved line-by-line coding to capture the underlying “meanings” in participants’ messages, rather than focusing solely on the words used.^37^ An iterative process was employed to identify common subthemes across codes, moving back and forth to uncover patterns and shared meanings. Figure 3 presents the key themes and subthemes.

In Phase 3, the findings were evaluated against the TTIH model to examine the relationship between existing and emerging digital technologies and their role in enhancing or transforming current services. This model was used to categorize the potential of new technologies, especially those capable of providing novel services. GS coded transcripts to develop the initial coding framework, which was reviewed by HD. The remaining transcripts were then coded according to this framework by GS and HD.

## Findings

A total of 29 interviews (*n* = 21) and two focus groups (*n* = 8) were conducted with 26 participants. This included 10 callers (all of whom were interviewed), nine supporters (three in a focus group and six in interviews), and nine stakeholders (four in interviews and a focus group with five participants). Three individuals were interviewed twice, at both the start and the end of the project. Table 1 displays the participation of target groups across the TPOM domains and data collection methods. Participant demographics (age) were not collected due to sensitivities around anonymity.

**Table 1.** Participation across target groups.

Participants	Main TPOM domains	Method	Participants	Interview or FG	Method	Sex
Callers	Technological social/human factors	Interviews	8	10 (two people interviewed twice)	Online and in person	Women = 4, Men = 4
Supporters	Technological, Social/human, and organizational factors	FG (1)	3	3	Online	Women = 2, Men = 1
		Interviews	6	7 (one person interviewed twice)	Online and in person	Women = 4, Men = 2
Community stakeholders	Organizational and wider macro-environment	FG (2)	5	5	In person	Woman = 3, Men = 2
		Interviews	4	4	Online	Woman = 3, Men = 1
Total number			26	29		

TPOM: Technology, People, Organizations, and Macro-environmental; FG: focus group.

Qualitative findings are presented under the main domains, in the “Technological aspects,” “People and human aspects,” and “Organization factors” sections.

### Here4UScotland app downloads and call volume

**Figure 4.**
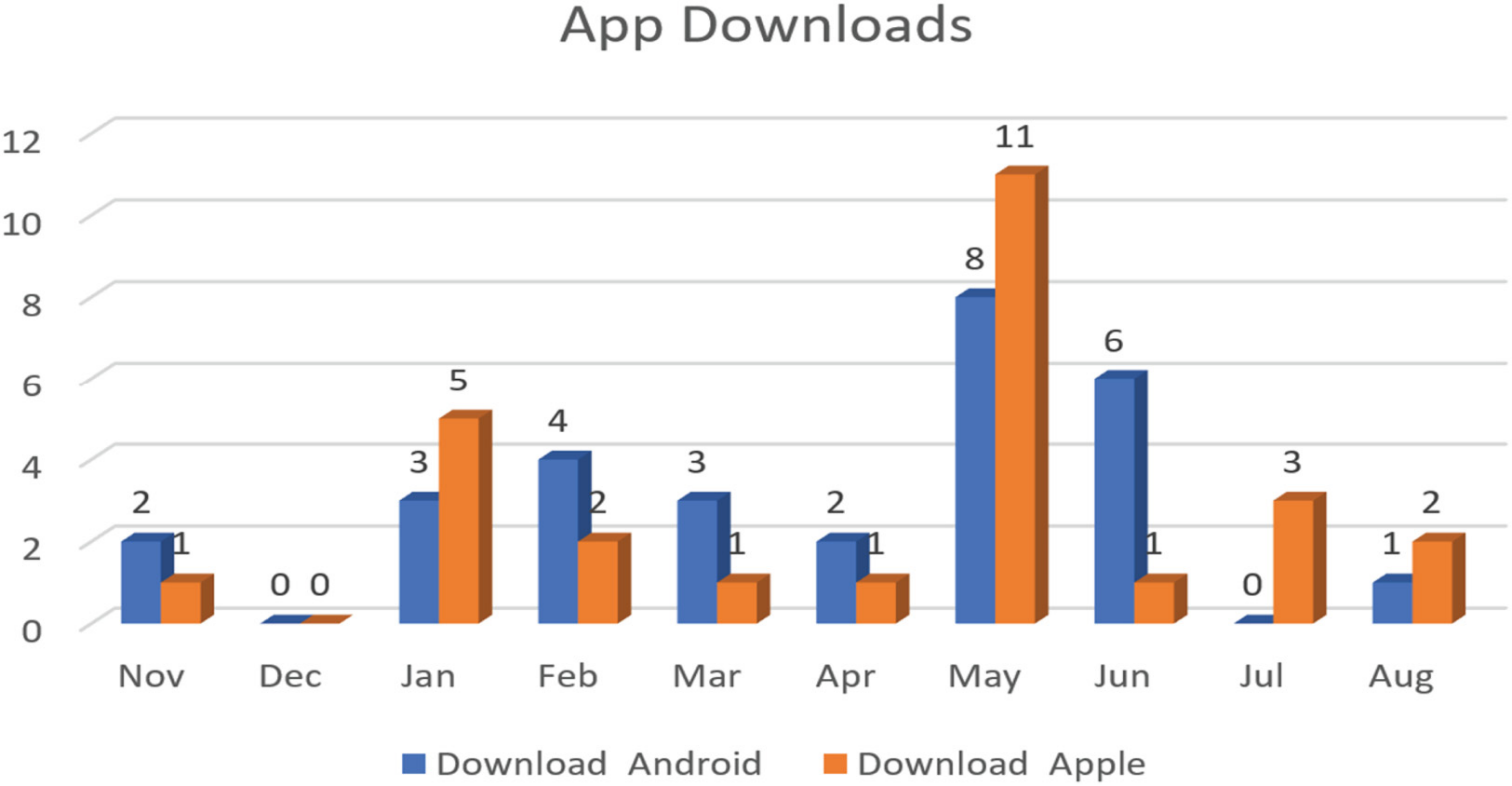
Number of app downloads until August 2023.

The Here4UScotland app piloted in Aberdeen, was operational from January to December 2023. For service users who did not have access to a smartphone, 25 smartphones (including prepaid credit for calls and Internet) were provided to ADA service users by DSL (funder of the project), along with the installed Here4UScotland app. Between January and August 2023, a total of 74 calls were logged (Figure 4).

**Figure 5.**
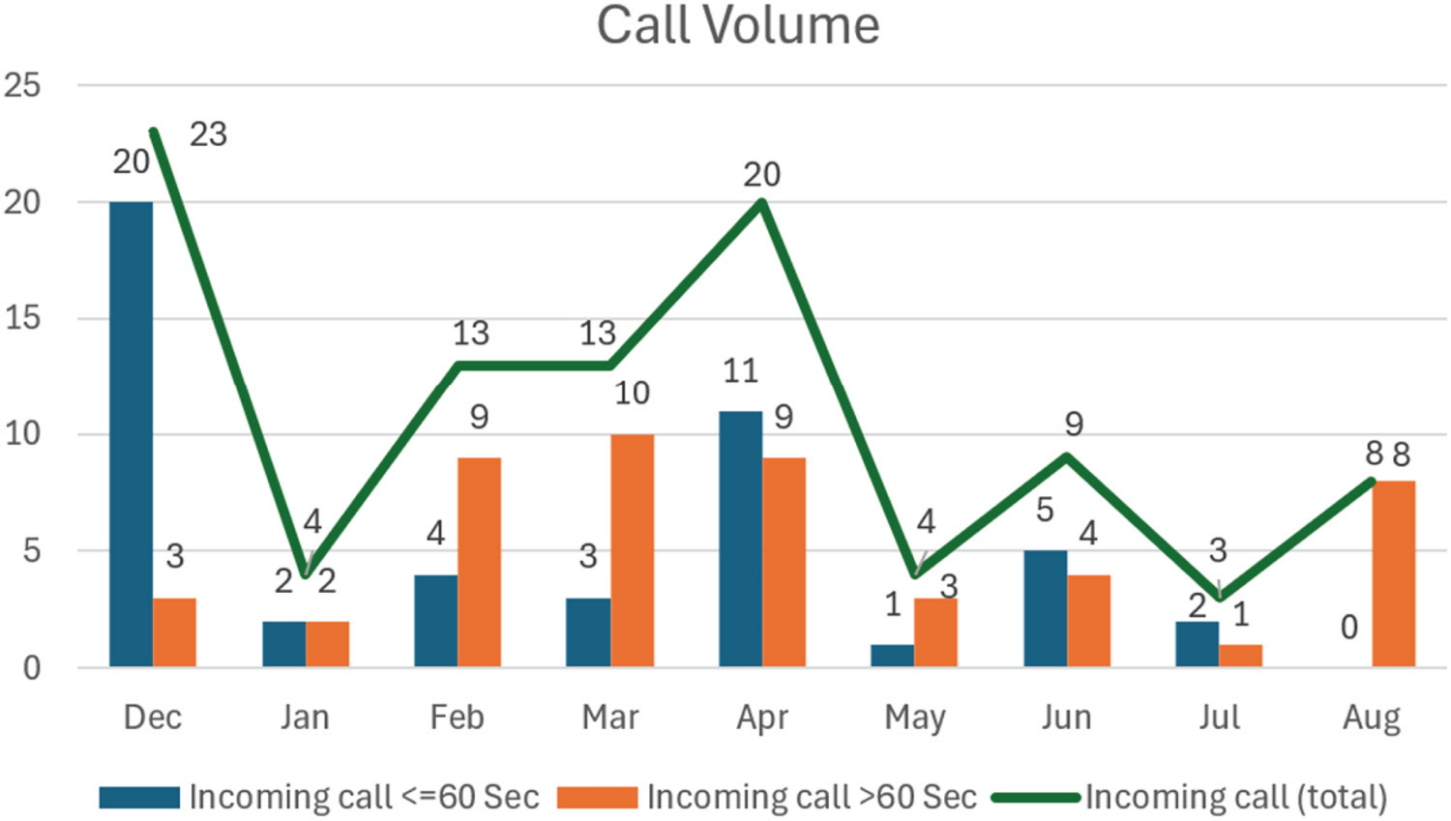
Call logs through August 2023.

Analysis of incoming calls to the app revealed trends in user engagement, with the highest activity recorded in April (Figure 5).

### Technological aspects

#### Accessibility and user comfort

Accessibility and user comfort were essential elements in the effectiveness of digital services for overdose prevention. Users valued systems that were easy to use and integrated seamlessly into their lives. For instance, location tracking features provided crucial real-time support, as noted:

Access to your location. Because the thing is we might personally know this person, he might be very unpredictable … You could see exactly where he is, you could send police. (Caller 6)

This capability was pivotal in emergencies, allowing rapid response to users in crisis.

However, some individuals expressed concerns over surveillance and the perceived intrusiveness of such technologies:

Yes, I was a bit dubious about this now … I got a little bit in my head about it, thinking oh, they’re tracking me. (Caller 2)

This highlighted a common tension between ensuring user safety and respecting privacy. To address these concerns, digital services needed to ensure that their features were effective and minimally invasive, promoting a user-friendly experience that did not compromise personal comfort.

#### Confidentiality and anonymity

Confidentiality and anonymity were central to the adoption and effectiveness of digital services in harm reduction, as callers often had concerns about how their personal and location data were handled. The emphasis on anonymization was evident:

So everyone's information can remain private on their phone … everything is anonymized. (Stakeholders–focus groups)

This showed a strong preference for systems that safeguarded personal information while providing essential services. However, there was also an openness to controlled data sharing when trust was established:

Once there's trust built with the system, people are willing to give up their privacy for short periods of time. (Stakeholders–focus groups)

Discussion around location tracking encapsulated the tension between its potential lifesaving benefits and the inherent risks associated with exposing personal whereabouts.

And if you want help to change your life then kind of you need parent figures in your life so for somebody to have control of your location for when you are not, ken [know], equipped to deal with life in itself without using drugs or putting yourself in danger, i.e., overdosing or even wanting to commit suicide. I think it's a horrible thing to do, ken [know]. (Caller 6)

This dynamic illustrated the complexity of implementing technology in sensitive contexts, necessitating clear policies and safeguards to protect user safety. This suggested that while users valued their anonymity, they were willing to engage more fully with the service if it proved trustworthy and beneficial.

#### Challenges and user suggestions

Implementing digital services for overdose prevention involved addressing several challenges. One key issue was the need for adequate support for both users and supporters.

Maybe giving people that option would be good … but supporters were potentially witnessing (graphic drug use) that there might need to be a bit more support for the supporters going forward. (Supporter 3)

This indicated a need for additional training and resources to help supporters manage their roles effectively, especially in high-stress situations. Another challenge was the integration of new features into existing systems. One respondent expressed concerns about the complexity:

Bringing in a third person to the call … just seems a bit bitty to me. (Supporter–focus groups)

To address this, services must ensure that new features are seamlessly integrated and provide continuous feedback from both callers and supporters.

Participants suggested notifications and alerts were vital components of digital overdose prevention services, providing users with timely information about potential risks. They proposed tailored notifications which could have warned users about dangerous drug batches or overdose risks locally:

If you get a notification, like say for example, hypothetical, I’m a heroin addict … I can get a notification saying, “Right, there's a bad batch in your area.” (Caller 1)

Developing a trustworthy and robust system for disseminating alerts was essential to ensuring that these notifications effectively enhance overdose prevention. Participants encouraged the integration of bespoke and reassuring video calls, representing a significant advancement in support provision. As one respondent highlighted:

Eye-to-eye contact is better than a chat on the phone. (Supporter 4)

Visual cues could have enhanced the effectiveness of support by cultivating a more nuanced understanding of the user's state. Nonetheless, the implementation of video calls comes with challenges. One participant noted:

Video calls take up a huge amount of bandwidth … and we already have connectivity issues. (Stakeholder–focus groups)

Balancing the benefits of alerts and video interactions with practical constraints such as internet connectivity and ensuring user privacy was crucial. Solutions might have included offering alternative modes of communication and ensuring sufficient call handling capacity.

### People and human aspects

#### Accessibility and ease of communication

The transition to digital services has significantly enhanced accessibility and simplified communication for individuals at risk of drug overdose.

I would say it's easier now because—Like there's a lot of stuff you can do, maybe online, like on, what do they call it—Like the Zoom platform, or maybe service using means you can attend or whatever, that's online, so maybe someone who's not well might not leave their house as long as they’ve got a Zoom App or access to a handset or something, from a client's perspective. Like most people have got a phone now. (Supporter 5)

This quote highlights how digital communication tools offered a more approachable, secure, and immediate form of contact, especially during crises when making a phone call might have been overwhelming. For many users, digital tools represented a lifeline.

… it definitely had a lot to do with being able to lift my phone out my pocket and touch and instantly speak to somebody straight away, …, I think it's a massive benefit because definitely for me and for a lot of people I ken [know] [with] addiction ken, the craving comes when—you never ken [know] when it comes or problems come, ken [know], in your life. Sometimes you just need ken [know] somebody on the phone to you. (Caller 6)

This reflected how digital services were perceived as a resource that users could rely on during moments of vulnerability. However, not everyone was comfortable with technology and could have found engaging with digital services and devices overwhelming:

I’ve actually forgotten the main purpose of it. I don't know what I’m supposed to use it for anymore. I’ve forgotten. And I haven't bothered to—I don't know where to find the information anymore. I’m sorry. (Caller 8)

This illustrated the difficulties some users faced in navigating digital services, highlighting the need for better and recurring training and a more intuitive design to ensure tools were used effectively.

#### Transforming relationships and support structures

The shift to digital services, particularly apps for harm reduction, has transformed relationships between clients and support workers. Traditionally, these connections were limited to scheduled meetings or occasional phone calls.

I think it's easier for clients just to pop us a text message, or send us a voice note, rather than having to ring up and have a phone call. (Supporter 6)

This informal communication allowed clients to reach out easily, reducing barriers and enabling more frequent check-ins. Another caller highlighted the app's role in preventing relapse:

If I didn't have the app on my phone, more than likely I would have definitely used [drugs]. (Caller 6)

The real-time support and increased accessibility appeared to foster trust and openness, as reflected here:

They were really open and honest with me about what they were using. (Supporter 3)

Now, with digital tools, communication was more flexible and continuous, building stronger connections and providing real-time support.

### Organization factors

#### User engagement and support mechanisms

User engagement and support mechanisms were crucial for ensuring that harm reduction digital tools were effective. This theme revolved around the necessity for supportive interactions and available assistance to navigate and utilize digital services effectively.

We've discovered that a lot of it is in how they encounter that app and what kind of supports are there when they encounter the app. (Stakeholder–focus groups)

This quote underscored that user support—whether through direct side-by-side guidance or community-based initiatives—was essential for ensuring that users felt comfortable and confident in using these tools. Additionally, the concept of “echoes of safety” reflected how repeated and varied interactions with harm reduction services reinforced safety messages:

When callers interact with different points of harm reduction, knowledge and information and support, they are getting echoes of safety. (Stakeholder–focus groups)

This thematic insight suggested that continuous, multi-faceted engagement with harm reduction services helped build a comprehensive safety net, which was crucial for effectively supporting individuals who used drugs.

#### Challenges in implementing digital services

There were various barriers to the effective use of technology in harm reduction. These challenges include issues with volunteer engagement, limitations of existing digital systems, and the need for better outreach and advertising. Volunteer supporters were required to share and log information following app use in the supporter role, via a MS Teams channel.

One participant's frustration with the limitations of digital tools and training platforms highlighted a key challenge:

I find team [MS] Teams limiting. So, I cannot imagine that it was very easy to do, well, a consistent interest in the app when it was all rooted in interacting in [MS]Teams. (Stakeholder–focus groups)

This indicated that the platform's limitations might have hindered volunteer engagement and the effective promotion of the app, reflecting a broader challenge of ensuring that digital tools were user-friendly and supportive.

#### Building trust and ensuring safety

Building trust and ensuring safety were crucial for the successful adoption and use of digital services in harm reduction. This theme encompassed the need for digital tools to be perceived as reliable and integral to users’ safety routines.

It becomes just as important, as making sure that you set out your naloxone and unlock your door. (Stakeholder–focus groups) [in case of overdose]

Reflecting the broader global digital shift, this participant illustrated how digital tools should have become a regular part of daily safety and merged with traditional harm reduction practices. This reflected the need for, and trust in, digital evolution concerning regular safety routines.

The emphasis on trust and reassurance was further supported by the need for consistent and supportive interactions with users:

We talk about this a lot with the volunteer team, with the Brave app. (Stakeholder–focus groups)

Ensuring that users felt valued and reassured through their interactions with digital services was critical for their successful integration and effectiveness.

### Macro-environmental factors

#### Impact of police involvement on digital services

One of the key emerging themes related to the impact of police presence on the effectiveness of digital services aimed at preventing OD. The involvement of law enforcement in overdose situations appeared to create significant barriers to the trust and utilization of these services by the target population. For instance, as in Scotland, the Police are notified alongside an ambulance call, a participant highlighted that people are:

less likely to phone an ambulance because they’re thinking a police officer might turn up instead. (Stakeholder–focus groups)

Digital communication strategies, such as the use of invasive and unsolicited “pester” messages, were employed by police following some successful drug operations. Although according to one participant, the reception to these messages varied, some recipients responded negatively, while others expressed appreciation:

Occasionally, there will be one or two messages that we had back which said, “Thanks very much for letting us know.” (Stakeholder–focus groups)

This suggested that while digital messaging could have been an important tool in drug prevention, its effectiveness depended on responsible implementation and careful consideration of how such communications were perceived by the target audience.

#### Challenges of digital transformation in sustaining services

The rapid deployment and adaptation of digital services during public health emergencies, such as the COVID-19 pandemic, have shaped expectations for similar responses to the ongoing overdose crisis. One participant remarked,

If we have a public health emergency, like the overdose crisis … that should enable funds to be unlocked in a much less bureaucratic process. (Stakeholder–focus groups)

This emphasized the importance of digital transformation in enabling swift action, reducing red tape, and ensuring that lifesaving services were not delayed due to procedural inefficiencies. Another matter revolved around the financial and operational challenges of sustaining digital services aimed at preventing drug overdoses.

We didn't see any of the funds coming in until 2022 … which is taking up how much of my time, how much of my time is worth, you know, it's a ridiculous amount of time. (Stakeholder–focus groups)

This highlighted the need for reliable funding mechanisms to support the continuous operation and development of digital services. Moreover, the strain of maintaining these services on limited budgets can impede the overall success and reach of the initiatives.

### Evaluation of the provision of services using the TTIH framework

**Figure 6.**
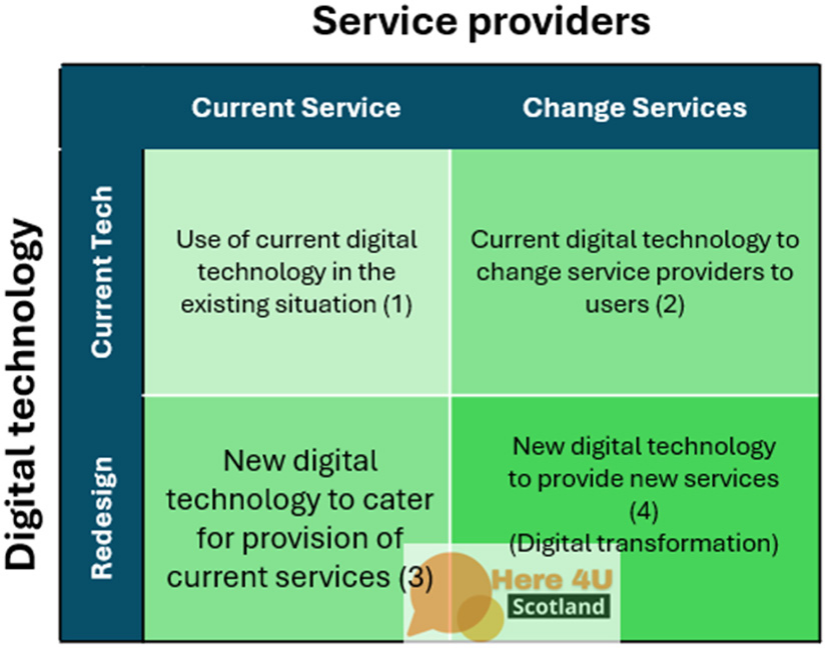
Here4UScotland in the transformative technology integration in health (TTIH) framework.

Our findings show that the Here4UScotland app, which is a re-design technology, demonstrates the provision of digitalized versions of current services as well as offering new services to its users. Figure 6 shows the examples of service provision using the Here4UScotland app.

The app enhances traditional services by providing remote supervision for individuals using drugs, thereby extending harm reduction strategies into digital spaces and increasing accessibility to critical support. Additionally, the app complements naloxone distribution programs by enabling timely interventions during overdoses, enhancing their efficacy through integrated digital tools. In developing new services, the app creates anonymized peer support networks, offering a stigma-free platform for individuals to connect and access assistance. Furthermore, the app collects anonymized data, providing valuable insights into drug use patterns, which can inform evidence-based public health strategies. Despite these advancements, challenges remain, particularly regarding privacy concerns, data security, and systemic barriers such as funding delays and resource limitations. Addressing these issues is essential to ensure the app's sustainability and broader adoption.

## Discussion

### Summary of findings

Key themes emphasized the importance of relationships, training, trust-building, strategic outreach, and implementation support in the effective delivery of emergency harm reduction digital services.

The discussion around technological aspects of overdose prevention highlighted the need to balance user accessibility, privacy concerns, and confidentiality concerns. Features like location tracking offer critical real-time support but also raise significant anonymity and privacy issues, necessitating a balance between providing lifesaving assistance and maintaining user confidence. Video calls, although advancing support with visual interaction, face challenges such as bandwidth limitations and privacy concerns. The suggested expansion of service models included broader harm reduction elements such as alerts and notifications, which would improve access to comprehensive support.

Organizational factors play a crucial role in the effectiveness of digital harm reduction services. Improving visibility and outreach through better marketing to those in need is essential. User engagement and support mechanisms are pivotal, with continuous assistance and community initiatives helping users navigate and utilize these tools effectively. Challenges such as platform limitations, volunteer engagement, and outreach inadequacies must be addressed to enhance effectiveness. Building trust in this way is crucial. Focusing on these organizational aspects could significantly improve the role of digital tools in preventing OD.

Macro-economic and environmental factors also impact the effectiveness of digital overdose prevention. The involvement of the police can deter individuals from seeking help. This reluctance underscores the need for strategies that maintain user trust and mitigate concerns about police involvement. Financial and operational challenges, such as delays in funding, can impede the deployment and maintenance of digital services. Innovative communication strategies, like targeted messages from police, show potential but require careful consideration of audience perception. Increasing visibility and offering incentives, such as vouchers, could further encourage the use and normalization of digital services like the Here4UScotland app.

### Contextualization of findings in the current literature

The proposed adoption of video and chat tools aligns with research on enhancing connection and monitoring during drug use.^38,39^ However, concerns surrounding their implementation underscore the need for a careful rollout of new technologies.

Findings on adaptability, flexibility, and accessibility highlight the importance of tailoring services to meet user needs, as emphasized in studies on context and usability.^40^ Desires for features like 24/7 access and key lock information highlight the necessity of accommodating diverse lifestyles.

Users’ perspectives on privacy mirrored broader concerns about digital security and surveillance.^41^ Nuances in the willingness to share location data reflect how trust, intentions, and perceived benefits influence user decisions. The preference for sharing data with trusted organizations highlights the critical role of confidentiality.

Openness to training and promoting buy-in align with best practices, such as comprehensive education and the use of success stories to enhance engagement.^42,43^ Relationships and trust are paramount, as strong connections with staff members foster user engagement.^44^ However, audio-only communication has been found to increase anxiety among supporters, indicating the need for multidimensional digital contact.^45^

Organizations have welcomed the digital evolution for information sharing and alerts. However, providing proper training and support for staff in crucial support roles is essential.^46^ Tailored debriefing and skills training can help staff manage the high stress associated with these roles.^47,48^ Strategic outreach is crucial for effectively engaging people who use drugs.^49^ Additionally, adequate resources, staffing, and careful planning are necessary for successful implementation.^50,51^

### Implications for digital transformation in harm reduction services

The integration of digital technologies into harm reduction services, exemplified by the Here4UScotland app, showcases the potential for innovative solutions in addressing DRDs. By applying TPOM and TTIH frameworks, the study highlights how features like real-time support, location tracking, and video interactions can enhance service accessibility and user engagement, while also offering new services.

However, the implementation of these digital tools also presents significant challenges. Privacy concerns, particularly regarding surveillance and data security, pose substantial barriers to user adoption. The study notes that while location tracking can provide critical real-time support, it also raises significant anonymity and privacy issues, necessitating a balance between providing lifesaving assistance and maintaining user confidence. This tension underscores the need for robust ethical frameworks to manage user data responsibly. To balance location tracking and privacy, some different methods were recommended, such as transparent consent, user-controlled settings, peer-led communication, user governance involvement, and clear crisis protocols. Alternatively, in the design section, co-design could be used to consider the users’ needs to build trust and enhance user engagement.

Additionally, the study identifies systemic barriers such as funding delays and resource constraints that hinder the sustainability of digital services. These challenges reflect broader issues in the digital transformation of health services, where financial and operational hurdles can impede the deployment and maintenance of innovative solutions.

The study's findings align with existing literature emphasizing that successful digital transformation requires not only technological adoption but also cultural and organizational readiness.^52–54^ Addressing these multifaceted challenges is crucial for maximizing the impact of digital interventions in harm reduction services.

### Strengths and limitations of the study

Here4UScotland was one of the first digital services aimed at supporting harm reduction to be introduced and evaluated in the UK, representing a pioneering effort to integrate technology into overdose prevention. A key strength of the research was the high level of engagement among participants, with good participation from both callers and supporters, despite the sensitive nature of the topic. This reflects the feasibility and acceptance of digital harm reduction services in a challenging area.

However, the research faced limitations, including the inability to interview participants who had direct experience with emergencies, which could have provided critical insights into the app's effectiveness during high-pressure scenarios. Additionally, resource and time constraints meant that the study involved an insufficient number of callers and supporters to evaluate the app's long-term sustainability comprehensively. These limitations highlight the need for further research with broader and more diverse participant groups to fully assess the scalability and impact of digital harm reduction services. On reflection, one of the supporter interview questions (Q#4) may be considered as leading—although in presenting, we tried to not lean to an answer. While the guide could not be modified retrospectively, we recognize this as a limitation and advise interpreting related findings with appropriate caution.

### Future research

Further research is needed to explore the digital adoption of such new services, particularly in the context of cultural or country-specific differences, with a focus on their implementation in the UK. Research could also explore the impacts of comprehensive communication systems in these contexts.

## Conclusion

The introduction of the app marked a shift toward integrating digital tools with traditional care models, recognizing that digital services offer new ways to engage with clients, especially those who are isolated or hesitant to seek help in person. However, there are concerns about digital tools being perceived as intrusive, impersonal, or inadequate compared to face-to-face support. Striking a balance between digital and traditional methods is crucial for success.

The integration of digital tools into harm reduction strategies has transformed support delivery and experience, enhancing accessibility and communication. Mobile apps and digital platforms provide an immediate, potentially less intimidating way for individuals to seek help in crises and prevent relapse, strengthening the relationship between users and support networks. Despite these advancements, challenges remain in user navigation of digital tools under stress, underscoring the need for clearer instructions and intuitive design. This shift from traditional face-to-face support to flexible, harm-reduction approaches reflects broader cultural and ideological changes in the field.

## Supplemental Material

sj-docx-1-dhj-10.1177_20552076251390561 - Supplemental material for Digital transformation of the harm reduction sector—“Here4UScotland” a case study of a virtual supervised consumption Supplemental material, sj-docx-1-dhj-10.1177_20552076251390561 for Digital transformation of the harm reduction sector—“Here4UScotland” a case study of a virtual supervised consumption by Hadi Daneshvar, Graeme Strachan and Catriona Matheson in DIGITAL HEALTH

sj-docx-2-dhj-10.1177_20552076251390561 - Supplemental material for Digital transformation of the harm reduction sector—“Here4UScotland” a case study of a virtual supervised consumption Supplemental material, sj-docx-2-dhj-10.1177_20552076251390561 for Digital transformation of the harm reduction sector—“Here4UScotland” a case study of a virtual supervised consumption by Hadi Daneshvar, Graeme Strachan and Catriona Matheson in DIGITAL HEALTH
