# Supplementary material for: Digital transformation of the harm reduction sector—“Here4UScotland” a case study of a virtual supervised consumption
Source: Digit Health. 2026 Jan 27;12:20552076251390561. doi: 10.1177/20552076251390561 (PMC12847686; doi:10.1177/20552076251390561)
Supplement: sj-docx-1-dhj-10.1177_20552076251390561 - Supplemental material for Digital transformation of the harm reduction sector—“Here4UScotland” a case study of a virtual supervised consumption [file sj-docx-1-dhj-10.1177_20552076251390561.docx]

**Appendix 1 – Topic Guides**

**Focus Groups Topic Guide - Supporter**

1. How did they become an app supporter? What was your motivation?
2. Have you been involved as a supporter for someone using the app?
   1. Yes: Has any overdose situation that happened during the call? Did the app work? Did you receive any support during/after the call?
   2. No: Why? Did not receive any calls?
3. Have you received any calls for just talking or providing information? Explain if yes.
4. In your opinion, has the app enabled supportive relationships to develop between callers (app user) and supporters (you)?
5. What services do you receive from the app and how do you feel about using it in terms of:
   1. Usability (how easy is it to use the app), learnability (e.g. how easy is it to learn to use the app), security, accessibility, confidentiality, ease of use, etc.
6. What benefits have you experienced from using the app so far?
7. When you started as a supporter and did you receive any training?
   1. If yes, how did you find out about it?
   2. If not, would you like to receive training? In which part?
8. Is the app sufficiently reliable?
9. What on the app do you think worked well?
   1. What did not work well?
   2. Prompt to expand on why if required
10. Do you have any negative/difficult experiences with the app?
11. Do you think, you need to receive any support? if yes, what kind of support do you need? (prompt: support for talking, technical support, support for dealing with PWUD, etc.)
12. In your opinion, what functions could be removed?
    1. What functions could be added?
13. Would you recommend this app to others as supporter?
14. Are features and functionalities implemented and used as you expected?
15. Is there anything else you’d like to add?

**Focus Groups Topic Guide - Community stakeholders**

1. Can you please tell me about how you involved service users in the programme (if applicable)?
2. What are your feelings about the services offered by the app in terms of:
   1. Usability / Learnability / Security / Accessibility / Confidentiality, etc.
3. What were the initial user reactions towards the services offered?
   1. Prompt: what did they find positive?
   2. What did they find negative?
4. Did perceptions change as they used the services? If so, how did they change?
5. What are the different uses of the app that you have come across?
   1. Prompt:– e.g. communication, information on harm reduction or service access, etc.?
6. What types of benefits have you identified from the use of the app so far?
7. What types of challenges have you identified from the use of the app so far?
8. Were there any unintended consequences in the use of the app services, either positive or negative?
9. What worked well in terms of the app? why?
10. What did not work well in terms of the app? Why?
11. Does the app change relationships between you and service users?
12. Would you recommend the use of the app to other users? Why?
13. In terms of the pilot of the app:
    1. Are management structures adequate to support services offered?
    2. Is the training provided adequate, realistic and effective?
    3. Are resources provided (including technology, change management and maintenance) adequate?
    4. Has the app changed the service for the user?
14. Would you like to see this app developed further? Prompt: If so, in what way? E.g. addition of new services etc.
15. Do you think this fits into the broader service provision landscape in the area to reduce drug harms?
16. Would you like further training on specific topics? (Prompt: drug awareness, digital technology and services)
17. Is there anything you would have wanted to be done differently?
18. Is there anything else you’d like to add?

**Interview Topic Guide - Supporter**

1. How did you become an app supporter? What was your motivation?
2. Have you been involved as a supporter for someone using the app?
   1. Yes: Has any overdose situation happened during the call? Did the app work? Did you receive any support during/after the call?
   2. No: Why? Did not receive any calls?
3. Have you received any calls for just talking or providing information? Explain if yes.
4. In your opinion, has the app enabled supportive relationships to develop between callers (app user) and supporters (you)?
5. What services do you receive from the app and how do you feel about using it in terms of:
   1. Usability (how easy is it to use the app), learnability (e.g. how easy is it to learn to use the app), security, accessibility, confidentiality, ease of use, etc.
6. What benefits have you experienced from using the app so far?
7. When you started as a supporter and did you receive any training?
   1. If yes, how did you find out about it?
   2. If not, would you like to receive training? In which part?
8. Is the app sufficiently reliable?
9. What on the app do you think worked well?
   1. What did not work well?
   2. Prompt to expand on why if required
10. Do you have any negative/difficult experiences with the app?
11. Do you think, you need to receive any support? if yes, what kind of support do you need? (prompt: support for talking, technical support, support for dealing with PWUD, etc.)
12. In your opinion, what functions could be removed?
    1. What functions could be added?
13. Would you recommend this app to others as supporters?
14. Are features and functionalities implemented and used as you expected?
15. Is there anything else you’d like to add?

**Interviews Topic Guide – Callers (App Users)**

1. Have you installed the app on your phone, and if so, have you used it so far?
   1. Installed/not used,
      1. Can you tell me why you did not use it?
      2. Have you tried to use it?
      3. What might help you use the app (device, remember, etc)?
   2. Installed/used
2. Can you tell me for what purposes you used the app? (prompt: for e.g. communication, safe use, etc)
3. Have you been involved in an overdose situation when you used this app? If so, please explain if this worked/ did not work in the situation.
4. Have you received training? If not, would you like to receive training?
   1. What type of training would you find useful – specific parts of the app or general use? (prompt: face-to-face, online video, etc.)
5. How do you feel about the app in terms of:
   ease of use and learning how to use, accessibility (initial setup, etc), security and confidentiality (date security, share your identity or identifying callers) , etc.
6. How do you/would you feel about the app having access to your location?
7. Have there been any benefits for you from your experience using the app?
8. What functions worked well for you in the app?
   1. Why did you like this function(s)?
9. What functions worked less well?
   1. Why did you not like this function(s)?
10. Do you have any negative/difficult experiences related to the initial setup or using the app?
11. Which functions do you think could be added to the app?
12. What functions do you think could be removed from the app?
13. Are features and functionality implemented and used as you expected?
14. Would you recommend this app to others?
15. Is there anything else you’d like to add?
