# Supplementary material for: Digital transformation of the harm reduction sector—“Here4UScotland” a case study of a virtual supervised consumption
Source: Digit Health. 2026 Jan 27;12:20552076251390561. doi: 10.1177/20552076251390561 (PMC12847686; doi:10.1177/20552076251390561)
Supplement: sj-docx-2-dhj-10.1177_20552076251390561 - Supplemental material for Digital transformation of the harm reduction sector—“Here4UScotland” a case study of a virtual supervised consumption [file sj-docx-2-dhj-10.1177_20552076251390561.docx]

# COREQ Checklist – Here4UScotland Evaluation

## Domain 1: Research team and reflexivity

1. Interviewer/facilitator: Interviews were conducted by GS; focus groups by GS, HD, and CM.

2. Credentials: All authors hold postgraduate qualifications (MSc/PhD).

3. Occupation: Researchers are academics in health informatics and public health.

4. Gender: Research team included both female and male researchers.

5. Experience and training: Researchers have extensive experience in harm reduction, digital health, and qualitative methods.

6. Relationship established: Relationships were limited; recruitment was via Alcohol & Drugs Action (ADA). Rapport established at interview.

7. Participant knowledge of interviewer: Participants were informed of the researchers’ roles and the study objectives.

8. Interviewer characteristics: GS has lived experience; this was disclosed where appropriate. Other researchers have professional expertise in harm reduction.

## Domain 2: Study design

9. Methodological orientation: Thematic analysis guided by TPOM and TTIH frameworks.

10. Sampling: Purposive sampling of callers, supporters, and stakeholders.

11. Method of approach: Recruitment through ADA (callers and supporters), email invitations, and direct contact (stakeholders).

12. Sample size: 29 participants (10 callers, 6 supporters, 7 stakeholders).

13. Non-participation: A small number declined due to time or personal reasons; not formally tracked.

14. Setting of data collection: Interviews via phone, MS Teams, or in-person; stakeholder focus group in Aberdeen.

15. Presence of non-participants: No non-participants present during interviews/focus groups.

16. Description of sample: Participants were adults (≥18) with recent drug use (callers), supporters, and community stakeholders. Ages not collected to preserve anonymity.

17. Interview guide: Developed by HD and CM, informed by TPOM; see supplementary file 1.

18. Repeat interviews: were conducted with some participants (both callers and supporters) a few months after the start and again at the end of the project.

19. Audio/visual recording: Interviews and focus groups were audio-recorded with consent.

20. Field notes: Researchers kept brief notes following sessions.

21. Duration: Interviews: ~30–60 minutes; focus groups: ~60–90 minutes.

22. Data saturation: Data collection stopped when thematic saturation was reached.

23. Transcripts returned: Transcripts were not returned to participants due to anonymity concerns; however, all transcripts were verified by a researcher against the original audio files.

## Domain 3: Analysis and findings

24. Number of data coders: GS coded all transcripts; coding framework reviewed by HD; consensus reached by team.

25. Description of coding tree: TPOM domains used as primary coding categories; subthemes developed inductively.

26. Derivation of themes: Themes derived both deductively (framework) and inductively (emerging patterns).

27. Software: NVivo (version 12) used for coding and analysis.

28. Participant checking: Findings not returned to participants; discussions triangulated within team.

29. Quotations presented: Verbatim quotations used with pseudonyms (Caller, Supporter, Stakeholder).

30. Data and findings consistent: Quotations support themes; consistency demonstrated in analysis.

31. Clarity of major themes: Major themes clearly presented in Results section (Figures 5).

32. Clarity of minor themes: Minor themes (e.g., user engagement) reported and contextualised.
